# Subacute Cardiac Tamponade in a COVID-19 Patient Despite Negative Testing

**DOI:** 10.7759/cureus.29090

**Published:** 2022-09-12

**Authors:** Neil R Kumar, Shreyans Patel, Bridget Norwood

**Affiliations:** 1 Internal Medicine, Jackson Memorial Hospital, University of Miami, Miami, USA; 2 Internal Medicine, Montefiore Medical Center, Albert Einstein College of Medicine, Bronx, USA; 3 Internal Medicine, Miller School of Medicine, University of Miami, Miami, USA

**Keywords:** hemorrhagic, effusion, respiratory, tamponade, covid-19

## Abstract

COVID-19 infection has been documented to cause a wide range of symptoms including cardiac complications. We present a case of subacute cardiac tamponade in a patient infected with COVID-19 in the absence of respiratory symptoms; we also review the current literature on this rare sequela. Our patient is a 67-year-old man who presented to the hospital due to intermittent chest pain for three weeks. COVID-19 polymerase chain reaction (PCR) testing was negative two times. He had an outpatient echocardiogram that showed a moderate pericardial effusion about a week prior to the hospital presentation. On admission, a repeat echocardiogram showed a large pericardial effusion with tamponade physiology. Pericardiocentesis did not reveal a clear etiology of the hemorrhagic effusion but four days later, the patient was found to be positive for COVID-19 infection without any clear respiratory illness. Given the absence of other etiology and negative workup, cardiac tamponade was attributed to pericardial inflammation from this virus and our patient improved with colchicine and steroids. We, therefore, advise providers to consider COVID-19 as a cause of hemorrhagic, cryptogenic cardiac tamponade despite negative COVID-19 testing. We also review 42 additional reported cases of cardiac tamponade in patients infected with COVID-19. COVID-19 can cause cardiac tamponade even in the absence of pulmonary disease. This case and literature review highlight tamponade as a rare complication of COVID-19 and should be considered in the differential of any acute deterioration in this patient population.

## Introduction

COVID-19, caused by severe acute respiratory syndrome coronavirus 2 (SARS-CoV-2), is a respiratory illness that has been associated with a wide range of symptoms with varying severity. It has been well documented that this virus can cause cardiac complications independent of a patient’s baseline comorbidities including acute coronary syndrome, pericarditis, myocarditis, and arrhythmia [1]. Cardiac tamponade, a life-threatening condition, has been documented as a rare sequela of COVID-19 infection. We report a case of an elderly man who presented with subacute cardiac tamponade attributed to COVID-19 infection without significant concurrent respiratory symptoms. We also review the current literature on this rare complication of COVID-19 infection.

## Case presentation

Our patient is a 67-year-old man who initially presented to the emergency department with chest discomfort and intermittent dyspnea. This patient had a medical history significant for melanoma treated with radiation therapy, Barrett’s esophagus, and hyperlipidemia. These symptoms originally started about three weeks prior to presentation during which time SARS-CoV-2 polymerase chain reaction (PCR) testing was negative two times. The following week he had a stress test negative for ischemia but underwent an echocardiogram that showed a moderate pericardial effusion. He was sent home from the clinic with a course of non-steroidal anti-inflammatory drugs (NSAIDs) at that time. His symptoms persisted which prompted him to return to the emergency department.

**Figure 1:**
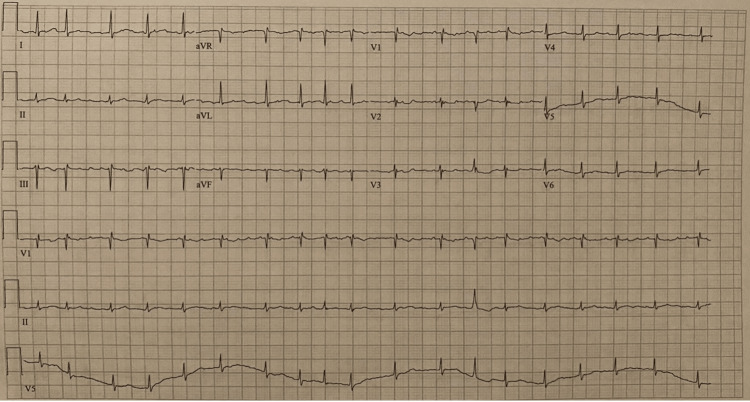
EKG on presentation showing new atrial fibrillation with rapid ventricular response as well as low voltage in precordial leads

**Figure 2:**
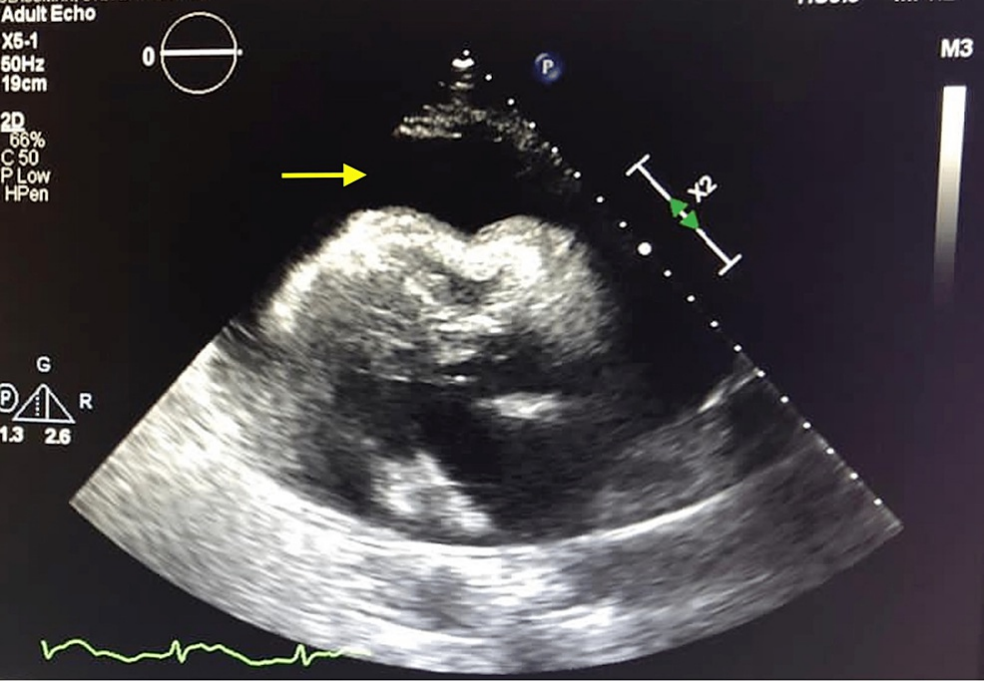
2D transthoracic echocardiogram on admission showing large pericardial effusion with diastolic collapse of right ventricle consistent with tamponade physiology

In the emergency department, presenting vitals and physical examination was unremarkable aside from tachycardia and distant heart sounds. Laboratory studies were significant for leukocytosis of 18.83 103/ul, C reactive protein of 31.25 mg/dl, normal electrolytes, and negative troponins. EKG was consistent with new atrial fibrillation with rapid ventricular response as well as low voltage in precordial leads (Figure 1). Bedside point of care ultrasound (POCUS) showed large circumferential pericardial effusion causing diastolic collapse of the right ventricle (Figure 2). Given the concern for early cardiac tamponade, the patient was taken for emergent pericardiocentesis with the removal of 750 cc of serosanguinous fluid. The patient was then started on colchicine as well as steroids and transferred to the intensive care unit for further monitoring.

**Figure 3:**
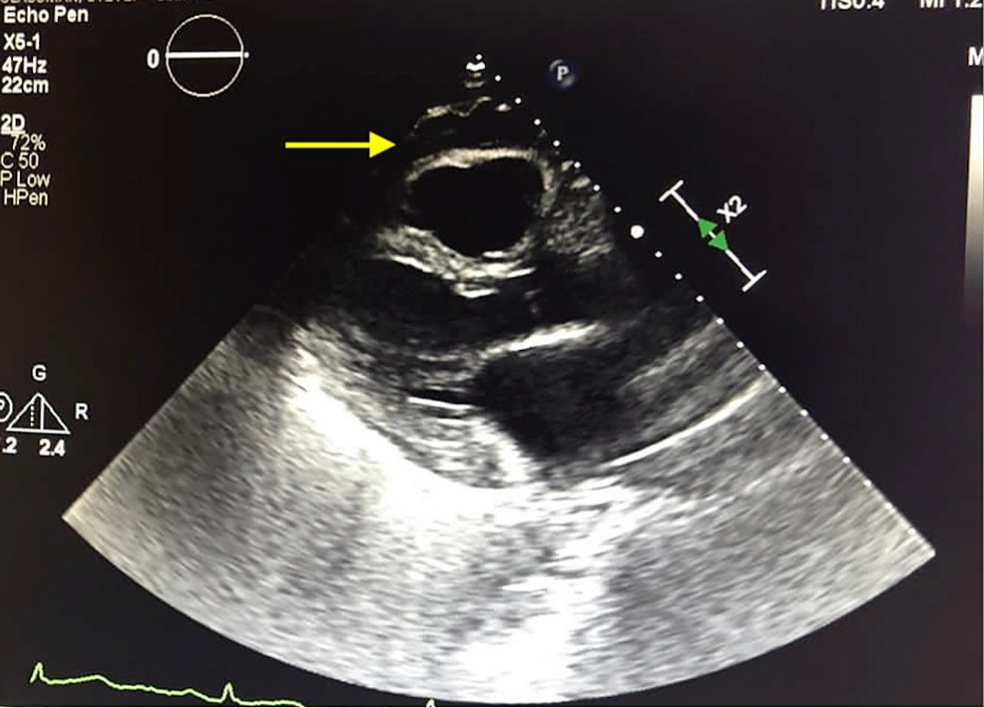
2D transthoracic echocardiogram post pericardiocentesis with removal of 750 cc of serosanguinous fluid

The patient reported improvement in symptoms and reverted to sinus rhythm without need for cardioversion. The pericardial drain was removed the following day without complication. Repeat echocardiogram at time of discharge showed no re-accumulation of pericardial fluid and normal left ventricular (LV) systolic function of 55% (Figure 3).

The etiology for pericardial effusion remained unclear at this time with viral pericarditis being the leading diagnosis even though viral panel, as well as SARS-CoV-2 PCR, was negative. Fluid from the pericardiocentesis was largely bloody with analysis showing 28,000 red blood cells/mm3 and 1,233 white blood cells/mm3 with 79% neutrophilic predominance. Fluid bacterial cultures, acid-fast stain, and autoimmune testing were all negative. Cytology and flow cytometry of pericardial fluid was also negative for malignancy. Pericardial fluid was not sent for SARS-CoV-2 testing. Due to clinical improvement, the patient was discharged home after a two-day hospital course with a regimen of colchicine and steroids for presumptive viral pericarditis.

**Figure 4:**
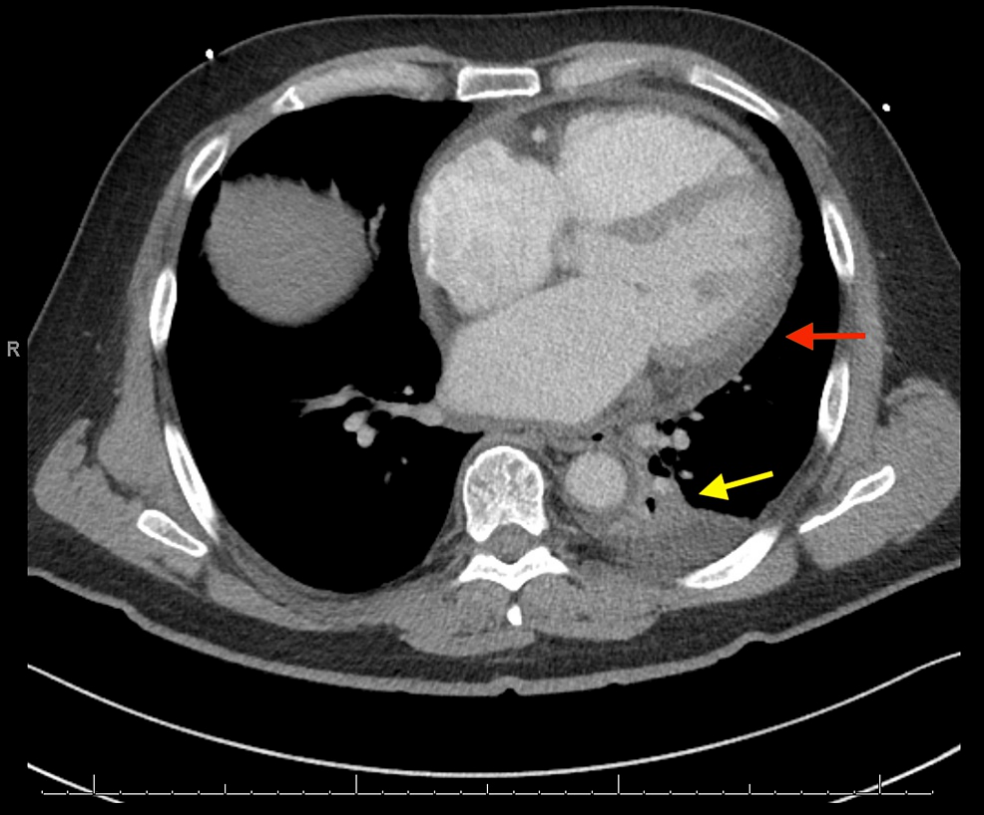
CT chest showing a small pericardial effusion as well as a left lower lobe infiltrate Red arrow refers to pericardial effusion; yellow arrow refers to left lower lobe infiltrate.

Four days later, the patient re-presented to the emergency department due to recurrent chest pain and persistent cough. Initial vitals and examination were unremarkable with oxygen saturation of 95% on room air. The patient tested positive for SARS-CoV-2 at this time by nasal PCR. Inflammatory markers were mildly elevated with lactate dehydrogenase of 287 units/L, C reactive protein of 3.67 mg/dL, and D-dimer of 3.05 ug/ml. Repeat echocardiogram showed a small pericardial effusion. CT Chest redemonstrated the small effusion, as well as a left lower lobe, infiltrate (Figure 4).

The patient was treated with colchicine as well as dexamethasone. He remained without any significant respiratory symptoms and was discharged home after a four-day hospital course with a negative PCR test result. The patient was followed up in the cardiology clinic a week post discharge where repeat echocardiogram showed minimal pericardial fluid.

## Discussion

Cardiac tamponade is a life-threatening condition that has a rare association with COVID-19 infection. The exact mechanism of cardiac injury by this virus is not well understood but is proposed to be due to the robust “cytokine storm” induced by the virus and the direct downregulation of myocardial ACE-2 receptors [1]. However, this pathogenesis is less likely in our case given the clinical picture and lack of elevated inflammatory markers especially C-reactive protein but could result from direct inflammation as usually in viral pericarditis. A meta-analysis of CT findings in patients infected with COVID-19 found that 4.55% of patients had evidence of pericardial effusion [2]. The clinical significance of this is unclear but may be related to myopericarditis induced by the virus. There have been several established cases including our patient that have documented the accumulation of pericardial fluid leading to tamponade physiology in patients infected with COVID-19.

Our case adds to a growing body of evidence that COVID-19 can lead to pericardial inflammation and cardiac tamponade independent of the patient’s cardiac risk factors. After careful literature review, we identified 44 other documented cases of cardiac tamponade in the context of COVID-19 infection (Table 1).

**Table 1:** Covid-19 Cardiac Tamponade AF - atrial fibrillation, AMS - altered mental status, BNP - brain naturetic peptide, CAD - coronary artery disease, CABG - coronary artery bypass graft, CK - creatinine kinase, CKD - chronic kidney disease, COPD - chronic obstructive pulmonary disease, CRP - C reactive protein, CRRT - continuous renal replacement therapy, CVA - cerebrovascular accident, DM - diabetes mellitus, ECMO - extracorporeal membrance oxygenation, ESRD - end stage renal disease, HF - heart failure, HFrEF - heart failure with reduced ejection fraction, HCQ - hydroxychloroquine, HLD - hyperlipidemia, HTN - hypertension, IVC – inferior vena cava, MV - mechanical ventilation, NSAID - non-steroidal anti-inflammatory drug, PPM - permanent pacemaker, RA - right atrium, RV - right ventricle, "-" refers to data that was not available

Patient	Age/Sex	Comorbidities	Presenting symptoms	Presenting exam	Inflammatory markers	Cardiac markers	Radiographic findings	EKG	2D Transthoracic Echo	Pneumonia	Mechanical ventilation	Management	Pericardial fluid	Outcome
1. current case	67 yo M	Melanoma, HLD	Chest pain, Dyspnea	Tachycardia	CRP-31.25 mg/dl	Troponin-negative	Unremarkable	Sinus tachycardia, Low voltage in precordial leads	Large circumferential pericardial effusion, RV diastolic collapse, LVEF-55%	None	None	Pericardiocentesis, Colchicine, NSAID	Bloody	Recovered
2. Hua et al. [3]	47 yo F	Prior myocarditis	Cough, Dyspnea, Chest pain, Fever	Hypotension, Tachycardia	-	Troponin T-0.225 ng/ml	Mild pulmonary congestion	Sinus tachycardia, Concave infero-lateral ST elevation	Global pericardial effusion, LVEF-Normal	None	None	Pericardiocentesis, Vasopressor	Serosanguinous	Recovered
3. Dabbagh et al. [4]	67 yo F	HFrEF (40%)	Cough, dyspnea, shoulder pain	Tachycardia	CRP-15.9 mg/dl, Ferritin-593 ng/ml, D-dimer-6.52 ug/mL	Troponin I-<0.018 ng/ml, pro-BNP-54 pg/ml	Unremarkable	Low voltage limb leads, Nonspecific ST elevation	Circumferential pericardial effusion, Early RV diastolic collapse LVEF-40%	None	Yes	Pericardiocentesis, HCQ, Colchicine, Steroids	Bloody	Recovered
4. Asif et al. [5]	70 yo F	CAD, DM2, HTN	Chest pain, Dyspnea	Fever, Hypoxia	-	-	Enlarged cardiac silhouette, Bilateral pulmonary infiltrates, Retro-cardiac opacities	Diffuse 1-mm ST-segment elevations, PR depression	Large circumferential pericardial effusion, RV diastolic collapse, Septal bounce, LVEF-55%	Yes	Yes	Pericardiocentesis, Vasopressor, Colchicine	Serosanguinous, Exudative	Recovered
5. Purohit et al. [6]	82 yo F	Paroxysmal AF, PPM, HTN	Cough, Fever, Chills	Unremarkable	-	Troponin-0.037 ng/ml	Significant circumferential pericardial effusion, Bilateral pleural effusions	A-paced rhythm, Diffuse T wave inversions	Circumferential pericardial effusion, Early RV diastolic collapse, LVEF-55%	None	None	Pericardiocentesis	Straw colored, Exudative	Recovered
6. Hakmi et al. [7]	48 yo M	Obesity, DM2	Dyspnea, Fatigue	Unremarkable	CRP-19.74 mg/dl	Troponin I-negative	Enlarged cardiac silhouette	-	Large pericardial effusion, Tamponade physiology	None	None	Pericardiocentesis	Yellow	Recovered
7. Hakmi et al. [7]	56 yo M	None	Cough, Chest pain, Fever, Chills	Hypotension	CRP-24.9 mg/dl	Troponin I-0.012 ng/ml	-	-	Large pericardial effusion, Tamponade physiology, LVEF-20%	None	None	Pericardiocentesis	Serous	Expired
8. Hakmi et al. [7]	55 yo M	Obesity, HTN	Cough, Fever, Chills	Hypotension	CRP-205.2 mg/dl	Troponin I-0.004 ng/ml	Bilateral lung opacities, Mildly enlarged cardiac silhouette	-	Large pericardial effusion, Tamponade physiology, Biventricular failure	Yes	Yes	Pericardiocentesis, Vasopressor, ECMO	Serosanguinous	Expired
9. Ruiz-Rodríguez et al. [8]	65 yo M	None	-	Hypotension, Hypoxia	Ferritin-0.3233 ng/ml, Fibrinogen-8.8 g/L, D-dimer-0.895 ug/ml	Troponin-0.192 ng/ml	-	-	3 cm pericardial effusion	Yes	Yes	Pericardiocentesis, Vasopressor, HCQ	Serous	Expired
10. Parsova et al. [9]	58 yo F	HTN	Dyspnea, Bilateral lower extremity edema	Tachypnea, Hypoxia, Tachycardia, Lung crackles	Unremarkable	Troponin T-0.00007 ng/ml	-	AF with rapid ventricular response, Low R voltage in the precordial leads	Circumferential pericardial effusion, Restricted diastolic filling, LVEF-30%	Yes	None	Pericardiocentesis	Serosanguinous, Exudative	Recovered
11. Torabi et al. [10]	42 yo F	Crohns disease, Guillain barré syndrome	AMS, Fever	Hypotension, Hypoxia, Tachycardia, Diffuse crackles	CRP-14.7 mg/dl Ferritin-310.1 ng/ml, D-dimer-2.26 ug/ml	Troponin-I-0.29 ng/ml, pro BNP-612 pg/ml	Patchy consolidative opacities	Low voltage in limb leads	Moderate pericardial effusion, RA systolic collapse, LV EF-20%	Yes	Yes	Pericardiocentesis, Intra-aortic balloon pump, Vasopressor	Serous	Expired
12. Singh et al. [11]	62 yo M	CAD w/ 1 stent, DM2, COPD, Obesity	AMS, Dyspnea	Hypotension, Hypoxia	D-dimer-2.90 ug/ml	Troponin-negative	Bilateral infiltrates, Right pleural effusion	Low voltage QRS	Large pericardial effusion, Tamponade physiology	Yes	Yes	Pericardiocentesis, Vasopressor, HCQ, Lopinavir-Ritonavir	Bloody, Transudative	Recovered
13. Dalen et al. [12]	55 yo F	None	Fatigue, Near syncope	Unremarkable	CRP-11 mg/dl	Troponin T-0.108ng/ml, pro-BNP-1025 pg/ml	Unremarkable	Sinus tachycardia, Insignificant ST-elevation in inferior leads, T-wave inversion in precordial leads, Low voltage	Large pericardial effusion, Tamponade physiology	None	None	Pericardiocentesis, Fluids, Vasopressor	Serosal	Recovered
14. Derveni et al. [13]	89 yo M	COPD	Dyspnea	Hypoxemia	CRP-24.77 mg/dl, Ferritin-227,900 ng/ml, D-dimer-1.65 ug/ml	Troponin-I-0.35 ng/ml	Bilateral lung infiltrates, Emphysema	Incomplete RBBB, New onset infero-lateral ST elevation	Anterior pericardial effusion, RV collapse LVEF-60%	Yes	Yes	Pericardiocentesis, HCQ, Azithromycin, Colchicine	Serous	Expired
15. Khatri et al. [14]	50 yo M	HTN, CVA	Cough, Dyspnea, Fever	Hypoxia	ESR-46 mm/hr, D-dimer-1.07 ug/ml, CRP-11.85 mg/dL, Ferritin-66,165 ng/ml	Troponin-0.544 ng/ml, CK-2135 u/l, CK-MB 54.3 ng/ml	Diffuse bilateral patchy opacities	Sinus tachycardia, ST-elevation in leads II, III, aVF, ST-depression in leads I, aVL	Large pericardial effusion with organizing material, Tamponade physiology	Yes	Yes	Pericardiocentesis, Vasopressor, IVIG	Serosanguinous	Expired
16. Walker et al. [15]	30 yo F	None	Fever, Cough, Chest pain	Tachycardia	D-dimer-0.26 ug/ml	pro-BNP-7890 pg/ml	Interstitial pneumonia, Subpleural interstitial densities and ground- glass opacities	Sinus tachycardia	12mm pericardial effusion	Yes	None	Pericardial window, Vasopressor, HCQ, Colchicine, Aspirin	Straw Colored	Recovered
17. Cairns et al. [16]	58 yo F	DM2, HTN	Fever, Diarrhea	Hypotension, Elevated JVP, Pulsus Paradoxus	Elevated	Troponin-0.3888 ng/ml	Bilateral chest consolidation	-	Large pericardial effusion, Tamponade physiology	None	None	Pericardiocentesis, Vasopressor	Serous	Recovered
18. Farina et al. [17]	59 yo M	CAD w/ CABG	Dyspnea, Chest Pain	Tachycardia	CRP-0.58 mg/dl, D-dimer-4.57 ug/ml	Troponin-I-22 ng/ml	“Ground glass areas," “Crazy paving pattern" in both lungs	-	Severe circumferential pericardial effusion, Collapse of the right heart sections	Yes	None	Pericardiocentesis, Lopinavir-ritonavir, HCQ	Hemorrhagic, COVID+	Recovered
19. García-Cruz et al. [18]	64 yo M	CAD	Chest Pain, Cough Fever	Hypoxia, Diffuse rales	-	-	Bilateral diffuse interstitial infiltrates	ST elevation in inferior and posterior leads	Pericardial effusion, Tamponade physiology	Yes	None	Pericardial window	Hemorrhagic	Recovered
20. Sauer et al. [19]	51 yo M	Asthma	Chest Pain, Dyspnea	Unremarkable	CRP-22.3 mg/dl	Troponin I-919 ng/ml	Moderate peripheral ground glass opacities, Voluminous pericardial effusion	Diffuse elevation of the ST segment, Low QRS voltage	Circumferential pericardial effusion, RV Compression	Yes	None	Pericardiocentesis, Colchicine	Hemorrhagic	Recovered
21. Sauer et al. [19]	84 yo F	HTN	Dyspnea, Fever	Decreased breath sounds, LE edema	CRP-6.6 mg/dl	Troponin-negative	Large, bilateral pleural effusion	-	Large pericardial effusion, Tamponade physiology	None	None	Pericardiocentesis, Colchicine	Serous	Recovered
22. Tiwary et al. [20]	30 yo M	DM1, CKDIII, HTN	Dyspnea, Abdominal pain	Hypoxia	CRP-8.9 mg/dl	Troponin I-0.09 ng/ml	"Typical changes consistent with COVID-19," R pleural effusion and pericardial effusion	Accelerated idioventricular rhythm	Large pericardial effusion, Early diastolic RV prolapse, Markedly thickened ventricular wall	Yes	Yes	Pericardial window, CRRT, Vasopressor	-	Recovered
23. Ejikeme et al. [21]	54 yo M	None	Chest Pain	Hypoxia	-	Troponin-negative	Cardiomegaly, Diffuse bilateral infiltrates	Non specific ST abnormalities	Large pericardial effusion, Decreased LVEF	Yes	None	Pericardiocentesis, HCQ, Steroids	Serosanguinous, Transudative	Recovered
24. Heidari et al. [22]	28 yo M	None	Chest Pain, Dyspnea	Hypotension, Tachycardia, Hypoxia	CRP-28.1 mg/dl. ESR- 90 mm/hr	Troponin-negative	Severe pericardial effusion, Left lower lobe collapse, Bilateral pleural effusion	Sinus tachycardia, Electrical alternans	Large pericardial effusion, RA inversion, RV diastolic collapse	None	None	Pericardiocentesis, NSAID, Colchicine, Lopinavir-Ritonavir	Hemorrhagic	Recovered
25. Gioia et al. [23]	57 yo F	HTN	Dyspnea	Hypotension, Tachycardia, Hypoxia	-	Troponin-I-64 ng/ml	Mild pulmonary congestion	Diffuse ST segment elevations	Moderate pericardial effusion	None	Yes	Pericardiocentesis, Vasopressor	Serous	Expired
26. Raymond et al. [24]	7 yo F	None	Chest Pain, Cough, Orthopnea	Tachycardia	CRP-5.11 mg/dL, ESR-43 mm/hr, Ferritin-134 ng/ml	Troponin I-0.01 ng/ml	Enlarged cardiac silhouette, Bilateral small pleural effusions	Sinus tachycardia, T-wave inversion in inferior and lateral leads, Low voltage QRS with electrical alternans	Large circumferential pericardial effusion, RA and RV wall collapse	None	Yes	Pericardiocentesis, NSAID, Colchicine, Pericardiectomy	Transudative	Recovered
27. Johny et al. [25]	30 yo M	None	Dyspnea, Orthopnea, Palpitations	Tachypnea, Tachycardia, Muffled heart sounds	-	-	Enlarged cardiac silhouette, Large left pleural effusion	Low voltage complexes	Large pericardial effusion, RA and RV diastolic collapse, Tamponade physiology	None	None	Pericardiocentesis, Colchicine, NSAIDs, Steroids, Antibiotics	Hemorrhagic	Recovered
28. Gill et al. [26]	34 yo F	None	Dyspnea, Chest Pain, Weakness	Tachypnea, Tachycardia, Cold extremities	Unremarkable	Troponin-0.55 ng/ml	Unremarkable	Low amplitude, PR depressions	Large pericardial effusion, RV diastolic collapse, Severe biventricular systolic dysfunction, LVEF- 20%	None	None	Pericardiocentesis, Colchicine, NSAID, ECMO	Serous	Recovered
29. Al-Kaf et al. [27]	21 yo M	Down syndrome	Dyspnea, Nasal congestion, Cough, Vomiting, Poor oral intake	Tachypnea, Hypoxia, Hypotension, Raised JVP, Distant heart sounds	CRP-5.2 mg/dl, D-dimer-2.0 ug/ml, Interleukin-6-130 pg/ml	Troponin T-0.043 ng/ml	Enlarged cardiac silhouette, Bilateral lung infiltrates	Diffuse low QRS voltage	Large circumferential pericardial effusion, RV diastolic collapse	Yes	Yes	Pericardiocentesis, Steroids, Heparin drip, Tocilizumab	Straw Colored, Exudative	Recovered
30.Mohammed Sheata et al. [28]	50 yo F	HTN, CKD	Fever, Cough	Tachypnea, Hypoxia, Hypertension, Tachycardia	CRP-15.9 mg/dl, Ferritin-1200 ng/ml, D-dimer-3.4 ug/ml	Troponin-0.149 ng/ml	Bilateral ground-glass appearance, Right sided pleural effusion, Enlarged cardiac silhouette	Sinus tachycardia, Diffuse low QRS voltage	Large circumferential, Pericardial effusion, RV diastolic collapse, Dilated inferior vena cava	Yes	Yes	Steroids, Vasopressor, Pericardiocentesis	Serous	Recovered
31. Gopal et al. [29]	40 yo M	CAD	None	Fever	Ferritin-195,321 ng/ml, D-dimer-8.03 ug/ml	-	-	Concave ST elevation in chest and limb leads, Reciprocal ST depression and PR elevation in aVR	Moderate pericardial effusion, Early signs of tamponade, Global biventricular dysfunction	None	Yes	Inotrope, Remdesivir, Steroids	Hemorrhagic	Expired
32. Gopal et al. [29]	49 yo M	CAD	None	Fever, Hypoxia	Ferritin-2,166 ng/ml, D-dimer-3.95 ug/ml	-	-	-	Pericardial effusion, Tamponade physiology	Yes	Yes	Remdesivir, Steroids, Inotrope	-	Recovered
33. Sampaio et al. [30]	45 yo F	None	Dyspnea, Fever, Myalgia	Tachycardia, Orthostatic hypotension, Tachypnea	CRP-2.1 mg/dl, Ferritin-478 ng/ml, D-dimer-0.543 ug/ml	Troponin I-0.867 ng/ml	Bilateral pulmonary infiltrates, Pleural and pericardial effusions	-	Moderate pericardial effusion, RV diastolic restriction	Yes	Yes	Antibiotics, Pericardial Drainage, ECMO, Vasopressors Tocilizumab, Steroids, Convalescent Plasma, Immunoglobulin	Citrine yellow	Recovered
34. Flores Cevallos et al. [31]	51 yo F	None	Syncope, Dyspnea	Hypotension	-	-	Bilateral infiltrates, Mild pericardial effusion, Pericardial thickening	Diffuse superior concave ST elevations	Pericardial effusion. Tamponade physiology, Deteriorated biventricular systolic function	Yes	Yes	Vasopressor, Pericardiocentesis	-	Recovered
35. Kogler et al. [32]	71 yo F	HTN	Chest Pain, Dyspnea	Tachycardia, JVD, Decreased heart sounds	-	Troponin T-0.14 ng/ml	Bilateral diffuse opacities	Low voltage	Moderate pericardial effusion, RV systolic compression, Paradoxical RV septal motion, End-diastolic RA collapse, Plethoric IVC	Yes	None	Fluids, NSAID, Colchicine, Steroids, Pericardiocentesis	-	Expired
36. Kogler et al. [32]	51 yo F	HTN, Obesity	Chest Pain, Dyspnea	Tachycardia Hypotension, Cold extremities	-	Troponin T-0.93 ng/ml	Bilateral patchy ground glass opacities	Low voltage, Diffuse ST elevations	Moderate effusion, Late RA diastolic collapse, RV compression, LVEF-20%	Yes	None	Fluids, Pericardiocentesis	Inflammatory, Exudative	Expired
37. Foster et al. [33]	44 yo F	Factor V Leiden deficiency, Pulmonary emboli, Hypothyroidism	Chest Pain	-	ESR-10 mm/hr, CRP-0.75 mg/dl, D-dimer-0.273 ug/ml	Troponin-0.4 ng/ml	Unremarkable	Borderline diffuse ST elevations, PR depression in leads II, III, AVF, mild PR elevation in aVR	Large pericardial effusion, RV diastolic invagination	None	None	Pericardial window, Colchicine	-	Recovered
38. Fox et al. [34]	43 yo M	None	Orthopnea, Dyspnea, Chest pain, Cough, Fever	Tachycardia, Hypoxia, Tachypnea, JVD, Pulsus paradoxus, Friction rub	D-dimer-6.32 ug/ml, Ferritin-1,077 ng/ml, CRP-36.8 mg/dl	Troponin-<0.006 ng/ml	Cardiomegaly	Low voltage, Diffuse concave ST elevations and PR depressions, PR elevation in aVR	Moderate circumferential pericardial effusion, Respiratory variation to LV inflow	None	None	Pericardiocentesis, Colchicine, NSAID	Serosanguinous	Recovered
39. Reddy et al. [35]	63 yo F	Myelofibrosis, Stem Cell Transplant, Graft-versus-host disease	Chest pain	-	CRP-5.9 mg/dl, D-dimer-0.743 ug/ml	Troponin-I-normal	Elevated right hemidiaphgram	PR depression, Saddle ST elevation in inferolateral leads	Large global pericardial effusion, RV diastolic collapse	None	None	Antibiotics, NSAID, Colchicine, Pericardiocentesis	Serosanguinous, Exudative	Recovered
40. Naderi et al. [36]	61 yo F	HTN, DM2, ESRD, Pacemaker	Dyspnea, Orthopnea Vomiting, Weakness	Hypoxia, Hypotension	-	-	Bilateral consolidations	Pacemaker rhythm	Massive pericardial effusiom	Yes	Yes	Vasopressor, Lopinavir/Ritonavir, IVIG, Pericardiocentesis	Exudative	Expired
41. Beckerman et al. [37]	55 yo M	HTN, Gout, Obesity	-	-	CRP-18 mg/dl, ESR-100 mm/hr	-	-	Low voltage, Nonspecific T wave changes in inferior leads	Circumferential pericardial effusion, RV collapse	Yes	Yes	Antibiotics, NSAID, Tocilizumab, Remdesivir, Convalescent plasma, Colchicine, Pericardiocentesis	Serosanguinous	Recovered
42. Deana et al. [38]	77 yo M	Chronic HF, HTN, DM2, COPD, CKD	-	Hypotension, Tachycardia	-	-	-	-	1.5cm pericardial effusion	None	None	Vasopressor, Pericardiocentesis, Steroid, Colchicine	Exudative, Inflammatory	Recovered
43. Schnaubelt et al. [39]	72 yo M	DM2, Persistent AF, Obstructive sleep apnea	Fever Fatigue	Bilateral crackles, Irregular heart rhythm, Hypoxemia Tachycardia	Elevated	Troponin T-0.08 ng/ml	Bilateral consolidations	-	2-3 cm pericardial effusion, LVEF-30%	Yes	Yes	Pericardiocentesis, Vasopressor, Steroids, Fluids	-	Expired
44. Darvishi et al. [40]	42 yo M	None	Chest pain, Diaphoresis, Dyspnea	Hypotension, JVD, Muffled heart sounds	-	Elevated	-	Acute extensive anterolateral STEMI	2 cm pericardial effusion, LVEF-20%	Yes	Yes	-	-	Expired
45. Sollie et al. [41]	29 yo F	None	Chest Pain, Dyspnea	Tachycardia. JVD, Distant heart sounds, Pulsus paradoxus	-	-	Pericardial effusion	Electrical alternans	>3.5cm pericardial effusion, RV diastolic collapse	None	None	Pericardiocentesis Aspirin, Colchicine, Steroids	Serosanguinous	Recovered

The first case of cardiac tamponade caused by COVID-19 was documented in early 2020 by Hua et al. in a 47-year-old female without any significant medical history. Of the total 45 cases examined, only 11 (24%) had any prior cardiac comorbidities with one patient having a prior history of myocarditis. There was no troponin elevation described in seven of the cases as well which suggests that this virus can mediate inflammation of the pericardium and accumulation of fluid without direct myocardial injury. Furthermore, 20 of the 45 patients did not have a concomitant pneumonia; in fact, 18 patients were not noted to have significant respiratory symptoms from COVID-19 infection and had primarily cardiac manifestations of this illness. Such observational data reinforces the premise that COVID-19 can cause significant pericarditis without respiratory involvement.natriuretic

Our patient proved to be a challenging diagnosis as on first presentation there was no clear etiology for the pericardial effusion. The only reported symptoms were intermittent chest pain and shortness of breath for three weeks without any other respiratory involvement or signs of infection. The differential included infectious process, malignancy given prior history of cancer, or autoimmune etiology; initial workup, however, was negative for any clear cause. We unfortunately were unable to send pericardial fluid for SARS-CoV-2 PCR testing. Our patient had a hemorrhagic pericardial effusion which has been demonstrated in some viral pericarditis, most prominently coxsackie virus [42]. However, hemorrhagic effusions have also been documented in the current literature on tamponade in COVID-19 patients and this patient had no other risk factors for a hemorrhagic effusion aside from remote history of malignancy for which cytology was negative. Our patient later tested positive for COVID-19 and we acknowledge the possibility that he could have been subsequently infected after the initial diagnosis of pericardial effusion. However, in absence of any other cause, direct COVID-induced pericarditis leading to pericardial effusion and tamponade was the most likely diagnosis.

Amongst the 36 patients with tamponade whose pericardial fluid was reported, 19 patients identified in this literature review were noted to have hemorrhagic or serosanguinous effusions on analysis after pericardiocentesis. Most commonly, hemorrhagic effusions are associated with malignancy, inflammatory states, or post infarction [43]. As mentioned above, viral pericarditis is typically noted to have a benign course, but there have been reports of hemorrhagic effusion most described in coxsackie virus infection where it is believed that the virus causes direct damage to myocardial cells or an immune-mediated injury [42]. Given the robust inflammatory response elicited by the COVID-19 infection and its cytokine storm, it may mediate hemorrhagic effusions through a similar mechanism. We urge providers to keep COVID-19 high on the differential when cryptogenic, hemorrhagic effusions of tamponade physiology are identified, even if repeat COVID-19 testing is negative.

Our patient presented with subacute cardiac tamponade as he had been experiencing symptoms intermittently for weeks prior to presentation. This case draws parallels to the patient described by Ejikeme et al. who presented with indolent symptoms and no hemodynamic compromise [21]. In fact, of the cases reviewed, only 16 (36%) presented with hemodynamic changes of hypotension and suspicion was only raised in other cases after echocardiogram showed a large pericardial effusion. This suggests that cardiac tamponade should be on the differential if a patient infected with COVID-19 experiences acute deterioration and hemodynamic compromise.

Management of cardiac tamponade is focused on prompt removal of the effusion and monitoring of hemodynamics post pericardiocentesis as well as volume resuscitation. One of the mainstays is to avoid positive pressure ventilation as increased intrathoracic pressure can impair cardiac filling [44]. This poses a problem in patients infected with COVID-19 as many require mechanical ventilation. Of the cases reported, 21 were on mechanical ventilatory support and 13 of those patients expired during hospitalization. Prompt evaluation using bedside US and drainage of pericardial fluid is of utmost importance in these patients presenting with cardiac tamponade.

Ultimately, our patient was diagnosed with cardiac tamponade due to viral pericarditis mediated by COVID-19 infection. The fact that he displayed little to no respiratory symptoms, no signs of myocardial damage, and initially tested negative for COVID-19 several times contributes to the uniqueness of this as a subacute presentation of tamponade. This case along with the others highlighted in this review document cardiac tamponade as a rare complication of COVID-19 infection.

## Conclusions

COVID-19 infection presents in many different ways and has been shown to affect a multitude of organ systems including the heart. We present a case of an elderly man with no cardiac comorbidities and minimal respiratory symptoms who presented with a very subacute cardiac tamponade caused by viral pericarditis secondary to COVID-19 infection. This case along with other well-documented reports included in this review highlight cardiac tamponade as a rare sequelae of this viral infection. We furthermore hope to inform providers to recognize COVID-19 as a considerable differential when encountering cryptogenic, hemorrhagic pericardial effusions of tamponade physiology, even without respiratory disease.
